# True bony TMJ ankylosis in children: Case report

**DOI:** 10.1016/j.ijscr.2019.06.037

**Published:** 2019-07-17

**Authors:** Rawaa Y. Al-Rawee, Ali Mohammad Saeed Al-Khayat, Saud salim Saeed

**Affiliations:** a Department of Oral and Maxillofacial Surgery, Al-Salam Teaching Hospital, Al-Sukar City, Mosul, Nineveh, Iraq; b Department of Oral and Maxillofacial Surgery, Al-Gemhory Teaching Hospital, Mosul, Iraq; c Department of Anesthesia and Intensive Care Unit, Al-Salam Teaching Hospital, Mosul, Iraq

**Keywords:** Tempromandibular joint trauma, TMJ ankylosis, Gap arthroplasty, Childhood trauma

## Abstract

**Introduction:**

The clinical importance of ankylosis in children is concerning with its massive effect and disturbance on the mandibular future growth causing gross deformity apart from the limited mouth opening. Trauma is the most common cause of bony and fibrous ankylosis. History, physical examination and radiographical examination of each patient with ankylosis of TMJ is mandatory in arriving to a final diagnosis, severity, involvement of adjacent structures and ultimately to plan the treatment.

**Case presentation:**

Ten years old girl presented with her family seeking solution for a severely limited mouth opening. From the history, the child has suffered from height fall at the age of 4 years. She had complained from swelling and pain near the ear, treated by analgesics, gradually subsided and neglected. This limitation affects on feeding and had an impact on the child's health, seeking for management for the condition became mandatory.

Clinical examination, radiographical examination is prepared in beside that ethical approval with full discussion with the parents done. Blind nasal intubation done.

**Discussion:**

Patients with true bilateral ankylosis are considered as the aggressive type. It’s not just because of ankylosis. Surgeons should keep in their minds that well experienced anesthetist is important; also extension of the ankylosed bone with amount of cutting is important and re ankylosis as a complication must be avoided.

**Conclusion:**

The surgeons agree with the statement that success in the preventing reankylosis after TMJ gap arthroplasty. Its primarily refers to the early postoperative physiotherapy, maintained on a long term.

## Highlights

• Ankylosis in children can affect growth, function, esthetic.
• Un observed or missed facial trauma in children can end with serious complication.
• Re-ankylosis is convoluted bothersome complication should kept in mind.
• Mouth opening should be at maximum range by the end of surgery.
• Patient follow up is mandatory for a period of time.

## 1 Introduction

Tempromandibular joint (TMJ) is a complex skeletal structure but it is important for normal functioning of the jaw also it's considered the most active functioning joint of the body. Essentially it has two joints with bilateral synovial articulation are connecting below with mandibular bone to the temporal bone of skull from above. Although of this bilateral articulation, these two joints work one unit so they are dependent of each other. TMJ is considered a unique type of joints in the body.

It is formed from the joint capsule, articular disc, mandibular condyles, and articular surface of the temporal bone, three ligaments, and lateral pterygoid muscles [1].

The growth center is located at the head of each mandibular condyle. Mandibular joint is unlike typical long bone. It is a multidirectional in its growth capacity. By time, the cartilage is replaced by bone. This growth center allows the increased length of the mandible needed for the larger permanent teeth, as well as affecting on all shape of the face. The growth center of bone within the condyle will disappear with full maturity [2].

Ankylosis can be defined as a stiffness of a joint due to abnormal adhesion and rigidity in bones of the joint.

It is means that the movement of condyle is limited which might lead to partial or complete inability to open the mouth. Mostly cases it is due to fusion (bony or fibrous) of the condyle of the mandible to the base of the skull [3].

The clinical importance of ankylosis in children is concerning with its massive effect and disturbance on the mandibular future growth causing gross deformity apart from the limited mouth opening.

Many classifications had been proposed for the ankylosis of the TMJ. It is based on location, type of tissue involving even extent of fusion. True complete bilateral intracapsular ankylosis was a bony fusion from the condyle to base of the skull reflects the most severe form.

According to the studies, a variety of factors can cause TMJ ankylosis like trauma (13%), local or systemic infection (53%) [4]. Systemic diseases such as Enclosing Spondylitis, Rheumatoid Arthritis and Psoriasis (28%). It occurs after TMJ surgery [5].

Trauma is the most common cause of bony and fibrous ankylosis [6]. Formation of intra-articular hematoma is followed by scarring with osseous replacement. It is believed to be the process of ankylosis following injury to the joint. This leads to narrowing of TMJ space [7]. Sometimes extension of fusion to the cranial base, sigmoid notch, zygomatic arch, coronoid process is seen in advanced cases [6].

History, physical examination also radiographical examination of each patient with ankylosis of TMJ is mandatory in arriving to a final diagnosis, severity, involvement of adjacent structures and ultimately to plan the treatment.

Computed Tomography (CT) had become the top imaging modality [3]. Relationship of the ankylotic mass with the middle cranial fossa, anteroposterior and mediolateral dimensions, and glenoid fossa can be assessed reliably and clearly in a series of very thin sections. For this reason it gives a very accurate and descriptive explanation in all three planes [5,6].

Creation of pseudoarthrosis aims to surgical intervention of the ankylosis. This will improve function or improving movement of the mandible [7,8], relieve airway obstruction if present [9,10] achieve normal growth and correction of deformity in children. It will restore appearance and occlusion in adults, prevent relapse and facilitate maintenance of good oral hygiene [7].

Surgical intervention is the main way to treat ankylosis. No single option for surgery has been shown entirely successful. Different surgeries had been described in the literature including condylectomy [7], simple arthroplasty [11], interposition arthroplasty [12] using temporal muscle, deep temporal fascia, fascia lata, ear cartilage or alloplastic material and reconstruction of the joint using costochondral graft (CCG), fibula, iliac, clavicle crest, metatarsal head or alloplastic material like acrylic or titanium prosthesis [13].

Relapse of ankylosis postoperatively is the most common complications. Its rate is as high as 50%. Many investigations believe that the choice of interpositional material is important in preventing recurrence [14].

A temporalis myofascial flap is used in maxillofacial reconstructive surgery [15].

The benefits of temporalis myofascial flap include close proximity to the joint which removes needing for additional surgery. It has adequate blood supply as an autogenous source. Inferiorly based temporalis myofascial flap is supplied with deep temporal artery (anterior and posterior branches), the terminal branches of the maxillary artery, as the main source of perfusion [16]. In comparison to costochondral graft is liable to fracture with over or under growth of the graft. Thus the temporalis muscle flap is the best choice to avoid recurrence

Most reported causes of reankylosis are inadequate removal of ankylotic mass [5,17] also, lack of compliance to post-operative exercises [18]. Anterior open bite occurred in patients treated with gap arthroplasty.

Lastly all the work has been reported in the SCARE Guideline Checklist [19].

## 2 Objective

To review the unexpected bad complications of trauma in children as well as draw attention with bilateral true bony ankylosis case report which managed by temporalis myofascial flap.

## 3 Case presentation

Ten years old child presented with her family seeking solution for a severely limited mouth opening. From the history, the child has suffered from fall from height at the age of 4 years. She had complained from swelling and pain near the ear, treated by analgesics, gradually subsided and neglected. The parents described the mouth opening had been reduced by time. When this limitation start to affect feeding and had an impact on the child's health, seeking for management for the condition became mandatory.

**Fig. 1:**
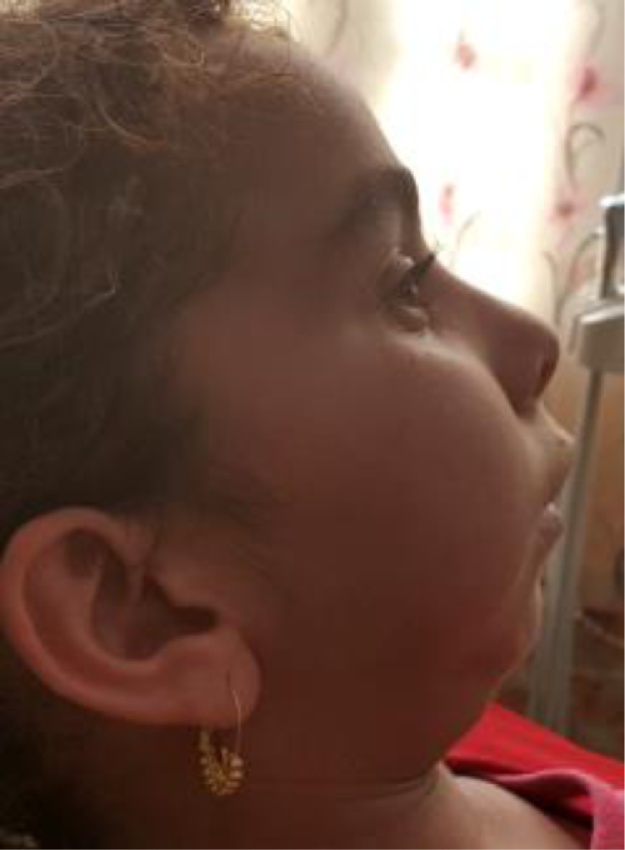
Clinical view of the child’s retruded mandible.

Clinical examination extraorally features looked pale, tired and psychologically upset child. It was obviously that she has retruded mandible, small chin with inability to protrude the mandible (Fig. 1). Intraoral examination showed class II relationship of occlusion, no mouth opening, multiple carious teeth and bad oral hygiene.

**Fig. 2:**
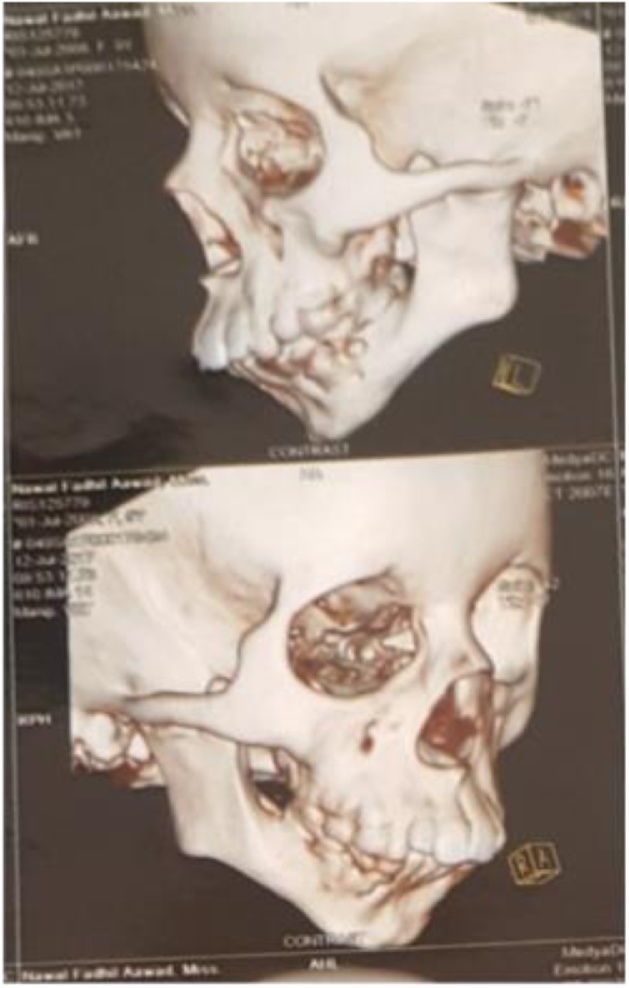
C.T. showing right and left side ankylosis.

Radiographical examination in orthopantomography was not conclusive for the tempromandibular joint area for this reason a computerized tomography (axial and coronal slices) was advised. Excellent visualization of the ankylosed joint on both sides was clear and sustained bilateral complete bony ankylosis (Fig. 2).

Preparation for management of the child started with a complete discussion of the treatment plan with the family. All difficulties and complications were explained to parents.

The patient underwent surgery under general anesthesia. Because of limited mouth opening, we need a blind nasal intubation or otherwise tracheostomy will take place with all its unwanted complications. In this particular case blind nasal intubation was done peacefully.

**Fig. 3:**
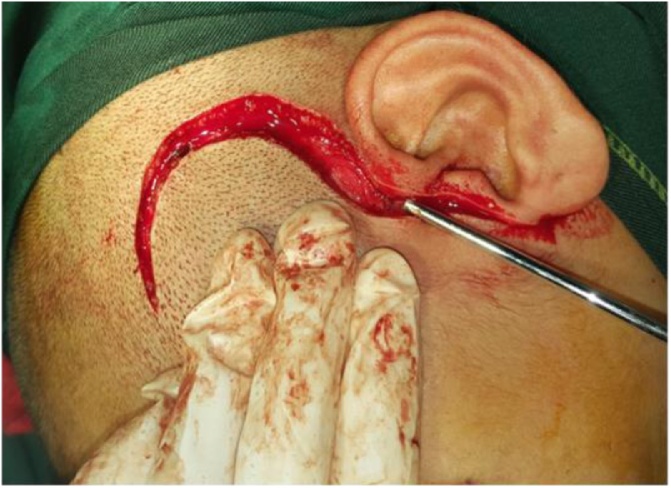
Preauricular incision was made as described by Al-Kayat and Bramley.

A Preauricular incision was extended inferiorly to the ear lobules and question mark incision extension superiorly to the hair line and was made as describing by Al-Kayat & Bramley (Fig. 3).

Dissection done in the blood less plane along with the external auditory meatus cartilage till the zygomatic arch root.

**Fig. 4:**
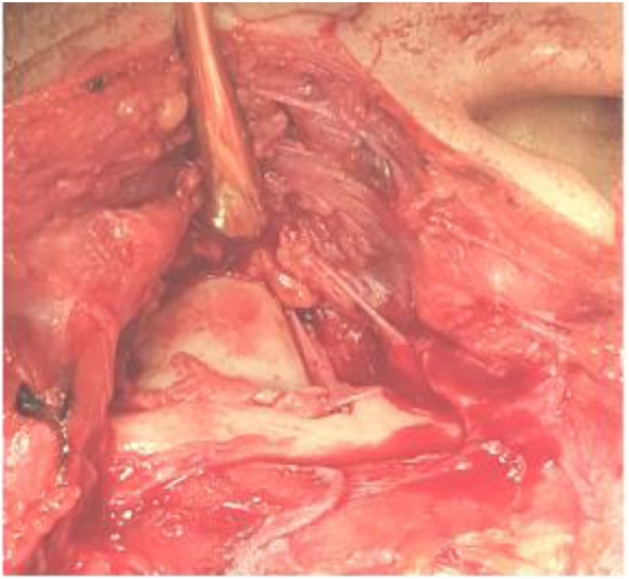
Exposure for the ankylosis after dissection.

**Fig. 5:**
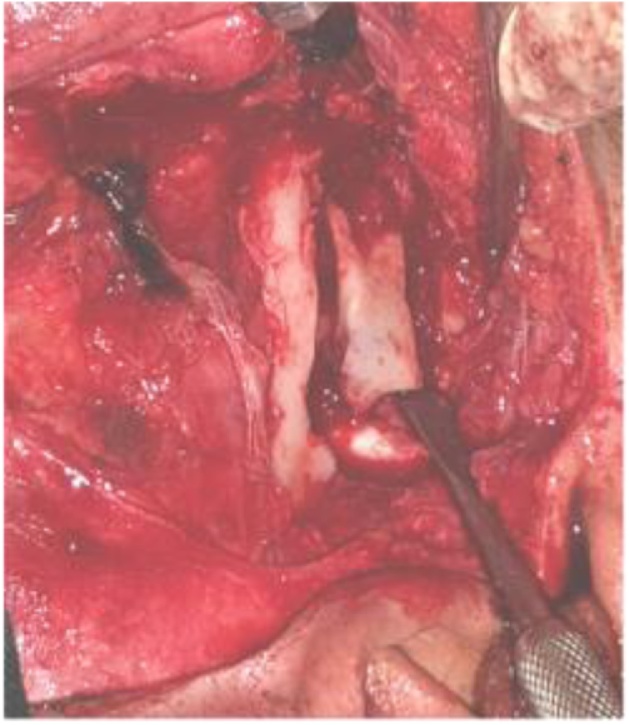
Ankylosed bony piece cutting.

Blunt subcutaneous tissue dissection was made on reaching the superficial temporal fascia in a question mark manner to expose the temporalis muscle. Then a superficial temporal fascia was incised and retracted anteriorly to protect the facial nerve branches. The periostium over the zygomatic arch was then incised sliding anteriorly by mucoperiosteal elevator and retracted with the superficial temporal fascia. TMJ capsule was clear (Fig. 4), T shapes incision done. The ankylotic mass involved both condyle and coronoid bone. Osteotomy was made to remove about 2 cm (Fig. 5). The excess of bone was removed with a large round bur. The same working was on the other side. A gap between the glenoid fossa and condyle was created taking care to maintain at least 4–5 mm of distance between the skull base and the real stump. Then the mandible mobilized to check it has a complete release.

**Fig. 6:**
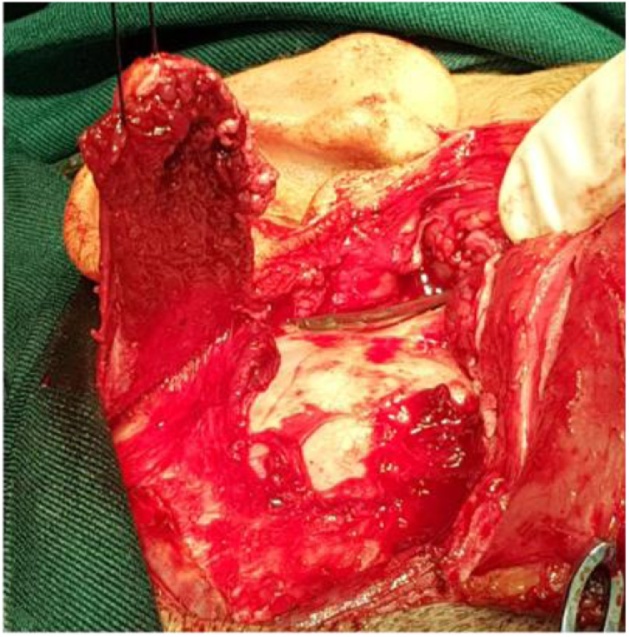
Temporalis muscle flap.

**Fig. 7:**
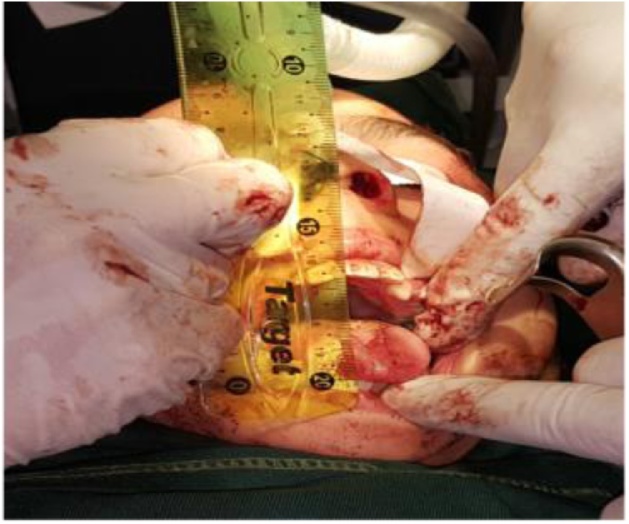
Intraoperative mouth opening.

A full thickness layer of temporalis muscle was harvested (Fig. 6) from the area posterior and superior to the ear. This is for avoiding any branches of the facial nerve, taking care not to harm the deep temporal muscle blood vessels. The graft was inserted below the zygomatic arch over the medial surface of mandible. It is pulled by suture to ensure the protection of reankylosis. The wound was then closed in layers. Mouth opening at the time of surgery was 40 mm (Fig. 7).

**Fig. 8:**
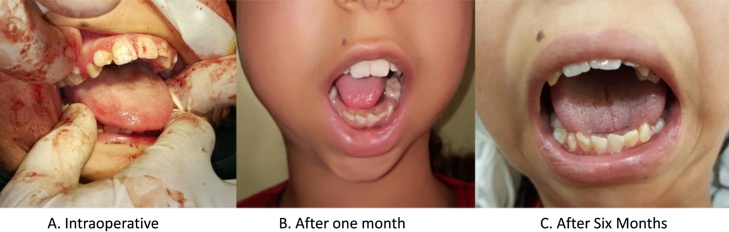
Mouth opening follow up.

Physiotherapy started after 4 days of surgery and maintained for 6 months. Maximum mouth opening is currently 35 mm. Close follow up took place with serial mouth opening checkup (Fig. 8).

## 4 Discussion

Maxillofacial surgeon knows that TMJ ankylosis is a rare condition [[20], [21], [22]], but its importance concerning with its massive effect in children specially. It affects on mastication, feeding, growth, articulation, and oral hygiene as well as general health.

Trauma considers the most common etiological factor. It is hypothesized that intra-articular hematoma, company with scarring. Formation of excessive bone, end with hypo mobility [23].

In children, different clinical scenarios are seen depending on the age at which ankylosis occurs, duration and on whether ankylosis is unilateral or bilateral.

No deviation, retruded mandibular bone, small chin, even nil mouth opening in the case presented here gave an idea that it is a bilateral problem. Limitation of mouth opening (1–2 mm) or nil opening in severe cases according to Graziani was considered as a bilateral bony type (true one). But Zarb et al. asserted that cases ranged with (5–7 mm) mouth opening with no protrusion or lateral movement were considered as true ankylosis [24].

Patients with true bilateral ankylosis are considered as the aggressive type, not just because of ankylosis, also the variable deformity in mandibular size and shape that need to be managed whether at childhood or in adulthood as seen in the case presented [3].

Computerized tomography (CT) has become the gold standard radiological investigation for managing patients with ankylosed TMJ [3]. Detect type, severity, extension, and relation is best showed by computerized tomographical slices. It is a mandatory examination.

Retrognathic mandible has specialized appearance in bilateral cases, both attenuated functional and aesthetic deficit; the condition has interference on speech, mastication and oral hygiene maintenance [25].

All cases of ankylosis should be managed surgically. Three clinical points most keep in mind.

The first is general anesthesia. Well experienced anesthetist is important. Limited mouth opening makes oral intubation missed. So blind nasal intubation must be performed otherwise tracheostomy should be done before starting surgery. Blind nasal intubation was done here by well experienced anesthetist avoiding the child all the unpleasant complications of tracheostomy that may follow.

Second critical point is extension of the ankylosed bone with amount of cutting done. In simple to moderate unilateral bony types condylectomy only can be beneficial to open the mouth. Bilateral true bony ankylosis needs condylectomy and coroinodectomy with good gap making to prevent reankylosis [26].

The interposition gap arthroplasty is the third critical point as reankylosis. It is the most common complication faced by the surgeon with post operative period. Many materials have been used to fill the gap [4]. Appropriate interpositional material includes:

1 Autogenous tissue: meniscus, muscle, fascia (temporalis muscle)
2 Allogeneic tissues; cartilage and durra.
3 Alloplastic: sialastic materials like acrylic, proplast, and silicon.

TMJ reconstruction materials are withdrawn because of many reasons [27] such as:

• Fascia lacks in volume
• Chondral graft shows resorption, infection and calcification
• Alloplastic implants have downside of corrode, break-up and cause foreign body giant cell reaction under functional loads.

As a result re-ankylosis, resorption, overgrowth, fracture, and pain are common complications with various autogenous and alloplastic materials reconstruction. Add to that the surgical site morbidity complications must keep in mind with TMJ reconstruction [4].

The temporalis muscle flap has been used for many years for restorations the gap. It is popularly used by surgeons because of its ease of handling, proximity to the temporal joint, good functional results, successful clinical results, with minimal complications [4], reliability, versatility, autogenous nature, resilience, adequate blood supply, ease of access to the condyle area, and ability to alter the arch of rotation by basing the flap inferiorly or posteriorly, allowing for a pedicle transfer of vascularized tissue into joint area. A satisfactory surgical outcome was obtained in the present case with the above method.

Patients with possible trauma to TMJ should be evaluated carefully by the clinicians. It needs to make an accurate early diagnosis.

Unnoticed trauma to the TMJ can cause potential complications concerning with the ankylosis even after several years later similar to the present case.

Recurrence is the most common disappointing complication as shown in literatures. For examples Lello one case from 13 was reankylosed [28], Guyuron and Lasa reported abnormal behavior of costochondral graft [29].

Recurrence percent were (7.1%) in Manganello-Souza and Miriani [30]. Ten percent (10%) [31]. In El-Mofty (1974). Rajgopal et al. achieved 100% success with gap arthroplasty [32]. Roychoudury et al., also reported good results of 50 cases treated with gap arthroplasty where reankylosis occurred in one patient (2%) [14].

The treatment of TMJ ankylosis through creating an adequate gap is of paramount importance in preventing any future recurrence and this can be achieved only when good access is gained to this complex anatomical joint [15,17].

Even in unilateral bony ankylosis lateral arthroplasty improved the facial pattern and MIO significantly when temporalis myofascial flap used as interpositional material [14].

These studies show that reankylosis is a common complication irrespective of surgical technique which is used treating the TMJ ankylosis.

Reiadh K. Al-Kamali in his original article concluded that the postoperative measurement of interincisal opening of ≥35 mm with lateral and protrusive movement was criteria for success of surgery. Reankylosis occurred in 7 unilateral and 12 bilateral cases from a total of 118 ankylosed joints. Postoperative jaw opening exercises are crucial for lasting success and failure of patient compliance is the cause of reankylosis [33].

Jaw-opening exercises must be done for months to years to maintain the surgical correction [34].

At last, a good surgery even co-operation of family with the child is mandatory for avoiding recurrence. Added to that physiotherapeutic exercises with good bone gap creation, and long follow up. These factors can play a role avoiding recurrence.

## 5 Conclusion

Success in the preventing reankylosis after TMJ gap arthroplasty is attached with early postoperative physiotherapy, maintained on a long term.

This describing technique is adequate bone removal and excellent intraoperative joint mobilization.

The temporalis myofascial flap is effective in treating TMJ ankylosis due to its reliability, versatility, autogenous nature, resilience, adequate blood supply, proximity to the joint, ease of access to the condyle area, minimal risk of nerve damage, and ability to alter the arch of rotation by basing the flap inferiorly or posteriorly, allowing for a pedicle transfer of vascularized tissue into joint area.

## Sources of funding

No funding sources.

## Ethical approval

Its approved by the Scientific Committee in Nenavah Health Directory.

Number: 7429. Date 14/3/201.

## Consent

Written informed consent was obtained from the patient’s parents for publication of this case report with accompanying images. A copy of the written consent is uploaded.

## Author’s contribution

Dr. Rawaa Y. Al-Rawee, Surgeon MaxFacs, Corresponding author, Writing, Prepare and responsible for submission.

Dr. Ali M. Saeed Al-Khayat, Drafting & Arrange Articles.

Dr. Saud salim Saeed. He is the Anesthetist. Great role in blind intubation.

## Registration of research studies

N/A.

## Guarantor

I am the Guarantor here Dr. Rawaa Y. Khaleel Al-Rawee.

## Provenance and peer review

Not commissioned, externally peer-reviewed.

## Declaration of Competing Interest

No conflict of interest for all authors in this paper.
